# From clinical judgment to biology: a clinician's opinion on the use of blood-based biomarkers in Alzheimer's disease

**DOI:** 10.3389/fneur.2026.1804163

**Published:** 2026-04-08

**Authors:** Raul Medina-Rioja, Aranza Llorente-Vidrio, Ramiro Ruiz-Garcia, Mayela Rodríguez-Violante

**Affiliations:** 1 Cognitive Neurology and Dementia Clinic, Instituto Nacional de Neurología y Neurocirugía “Manuel Velasco Suárez.” Tlalpan, Mexico City, Mexico; 2 Psychiatry department, Instituto Nacional de Neurología y Neurocirugía “Manuel Velasco Suárez.” Tlalpan, Mexico City, Mexico; 3 Clinical Neurodegenerative Diseases Research Unit, Instituto Nacional de Neurología y Neurocirugía “Manuel Velasco Suárez.” Tlalpan, Mexico City, Mexico

**Keywords:** Alzheimer, biomarker, dementia, diagnosis, Ptau

## Introduction

The diagnosis of Alzheimer's disease (AD) has undergone a substantial transformation over the past two decades. The shift from a purely clinical approach to a clinical–biological paradigm has enabled the integration of evidence supporting the clinical use of biomarkers in cerebrospinal fluid (CSF), neuroimaging, and more recently (and most prominently) blood-based biomarkers. Among these, phosphorylated tau at position 217 (p-tau217) has emerged as a marker with high sensitivity and specificity, approaching 85%−90%, for the detection of amyloid and tau pathology characteristic of AD (1), offering the promise of a more accurate diagnosis without the need for costly imaging studies or invasive procedures such as diagnostic lumbar puncture.

However, the incorporation of biomarkers into diagnostic practice is not without dilemmas: these range from the risk of overdiagnosis in cognitively healthy individuals to the difficulty of distinguishing AD from other dementias with mixed pathology. Furthermore, several variables, such as obesity and renal failure, can alter the sensitivity of blood-based assays (2, 3).

In this opinion, we propose that the current biomarker-driven paradigm risks promoting a biologically reductive approach to diagnosis, potentially leading to overdiagnosis and misclassification, particularly in cognitively unimpaired individuals. We aim to critically examine the clinical and biological roles of blood-based biomarkers and advocate for a balanced clinical–biological framework in which biomarkers inform, but do not replace, clinical judgment.

## The diagnosis of Alzheimer's disease

Traditionally, AD was diagnosed based on the clinical triad of progressive cognitive decline, insidious onset, and a hippocampal amnestic syndrome, characterized by impaired encoding of new information that interferes with activities of daily living. Neuropsychological assessment and the exclusion of secondary causes of dementia have classically been the pillars of diagnosis. Over time, neuropathological research and neuroimaging allowed this diagnosis to be anchored to objective findings: amyloid plaques and neurofibrillary tangles composed of tau protein (2).

Consequently, the concept of AD has expanded into a clinical–biological continuum, in which clinical features, neuroimaging, and biomarkers converge to provide diagnostic certainty. This shift reflects the need to detect the disease at earlier stages, design better clinical trials and to offer disease-modifying therapies, whose effectiveness largely depends on the timing of their initiation (3).

### Current diagnostic criteria

In 2024, diagnostic criteria underwent two major updates. The first was proposed by the Alzheimer's Association, which revised disease staging by adding a preclinical or asymptomatic stage in which biomarkers of AD can be detected in individuals without symptoms, largely dispensing with clinical features and focusing instead on the biological behavior of the disease (4).

In contrast, the International Working Group (IWG) advocated for a clinical–biological approach that requires the presence of a compatible clinical phenotype alongside positive biomarker evidence. According to this framework, individuals with positive biomarkers but preserved cognition should be considered only “at risk” and should not be diagnosed with AD. This position is justified by the fact that not all individuals with positive biomarkers will progress to symptomatic disease, as well as by the considerable variability in biomarker results depending on the type of marker and the laboratory performing the analysis (3).

Both frameworks agree that biomarkers enhance diagnostic certainty but differ in the degree of reliance placed upon them. For clinical practice, the IWG position appears more prudent: diagnosis should be based on clinical presentation plus biomarkers, rather than biomarkers in isolation. Although both frameworks are debatable, the central question remains: can (or should) we diagnose disease solely on the basis of a positive biomarker?

### Biomarkers of Alzheimer's disease

The development of biomarkers has enabled the *in vivo* correlation of amyloid plaques and neurofibrillary tangles with measurable biochemical changes, marking a major diagnostic shift in Alzheimer's disease. Historically, definitive diagnosis relied on autopsy, with clinical accuracy around 70% even in specialized centers. Autopsy studies have revealed significant clinicopathological discordance, reflecting the heterogeneity of dementia and frequent mixed pathologies. While biomarkers improve diagnostic precision, these findings highlight that uncertainty persists and reinforce the need for their interpretation within a clinical framework (4).

Currently available biomarkers include CSF measurements (Aβ42/Aβ40 ratio, total tau, and phosphorylated tau), PET imaging (amyloid), and more recently blood-based biomarkers (Aβ42/Aβ40 ratio, p-tau217, and p-tau181) (5).

Among these, p-tau217 stands out for its superior diagnostic accuracy compared with other epitopes such as p-tau181, achieving sensitivities and specificities close to 85%−90% for identifying amyloid and tau positivity, particularly when combined with Aβ42. Its performance has been such that the p-tau217/Aβ42 index has even received FDA approval for the diagnosis of the disease (1).

Blood-based biomarkers represent a major logistical advance, as they are less invasive and less costly, and reduce the need for diagnostic lumbar punctures or neuroimaging studies, which are expensive and have limited availability, thus seemingly facilitating the diagnosis of AD. Additionally, they have shown the ability to distinguish Alzheimer's disease from other dementias (1).

### False-positive scenarios

One of the main risks associated with blood-based biomarkers is that they may be elevated in other conditions. This implies that biomarker positivity does not automatically equate to AD, but rather reflects the presence of neuropathological processes that may coexist with or mimic the disease, potentially leading to misdiagnosis, unnecessary treatments, or delays in the diagnosis and management of other conditions. Moreover, there are well-described scenarios in which cognitively healthy individuals with positive biomarkers (up to 30% of adults) never develop clinical dementia. In such cases, an elevated p-tau217 level could incorrectly label an individual as having AD when they are merely at risk and may never develop the disease (3, 6).

Therefore, blood-based biomarkers are not infallible. These scenarios reinforce the need to interpret them within a clinical and syndromic framework, avoiding premature or incorrect diagnoses that may generate uncertainty for patients and families, stigmatization, or inappropriate diagnostic approaches or treatments.

### Prioritizing clinical assessment as the diagnostic axis

Despite the enthusiasm surrounding biomarkers, the neurological clinical examination, together with recognition of atypical variants (dysexecutive frontal, behavioral frontal, posterior cortical atrophy, logopenic aphasia, and corticobasal syndrome with Alzheimer pathology), comprehensive neuropsychological assessment, and correlation with neuroimaging, must remain central to the diagnostic process (3, 5, 6).

Biomarkers, including p-tau217, should be interpreted as adjunctive tools that increase diagnostic certainty, but they should never replace clinical judgment. Ordering biomarkers without a clear indication or overinterpreting their results may lead to false diagnoses of AD in healthy individuals or in those with a different primary etiology, with potentially significant consequences, particularly in the current era, where biological therapies are becoming increasingly relevant.

## Conclusion

Blood-based biomarkers represent a major advance in the diagnosis of Alzheimer's disease by providing accessible and accurate detection of underlying pathology. However, their isolated interpretation may lead to misclassification, particularly in individuals with mixed pathology or preclinical biomarker positivity. In this perspective, we emphasize that biomarkers should be integrated within a clinical–biological framework, where biological evidence supports—but does not replace—clinical assessment. The diagnostic process must remain anchored in clinical judgment, with biomarkers serving as complementary tools that enhance, rather than define, diagnosis. This approach mitigates the risk of overdiagnosis and reinforces the importance of preserving clinical reasoning in the era of biomarker-driven medicine.
